# Supplementary material for: Vitamin D Modified DSS-Induced Colitis in Mice via STING Signaling Pathway
Source: Biology (Basel). 2025 Jun 18;14(6):715. doi: 10.3390/biology14060715 (PMC12190092; doi:10.3390/biology14060715)
Supplement: Supplementary file 1 [file biology-14-00715-s001.zip › Supplementary Materials S1: 16s-report of gut microbiota/Groups/Rankabundance_curve/Rank_Abundance_Group.pdf]

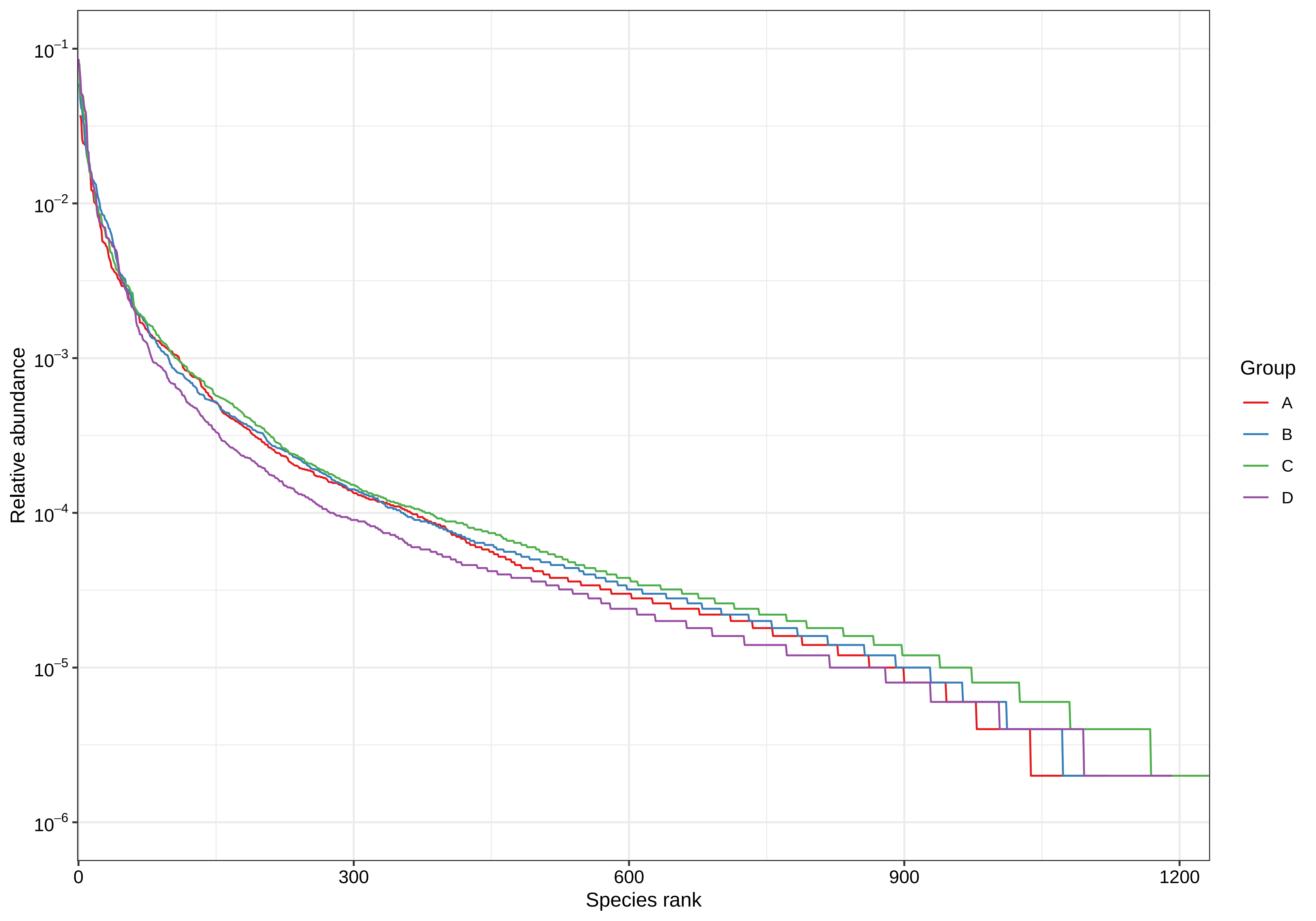
Relative abundance
10^{-1}
10^{-2}
10^{-3}
10^{-4}
10^{-5}
10^{-6}
0
300
600
900
1200
Species rank
Group
A
B
C
D
